# Skin and Systemic Inflammation in Schnitzler's Syndrome Are Associated With Neutrophil Extracellular Trap Formation

**DOI:** 10.3389/fimmu.2019.00546

**Published:** 2019-03-22

**Authors:** Hanna Bonnekoh, Jörg Scheffel, Jim Wu, Sheila Hoffmann, Marcus Maurer, Karoline Krause

**Affiliations:** ^1^Department of Dermatology and Allergy, Allergie-Centrum-Charité, Berlin Institute of Health, Charité – Universitätsmedizin Berlin, Freie Universität Berlin, Humboldt-Universität zu Berlin, Berlin, Germany; ^2^Autoinflammation Reference Center Charité (ARC2), Berlin Institute of Health, Charité – Universitätsmedizin Berlin, Freie Universität Berlin, Humboldt-Universität zu Berlin, Berlin, Germany; ^3^German Center for Neurodegenerative Diseases, Berlin, Germany

**Keywords:** Schnitzler's syndrome, neutrophil extracellular traps, neutrophils, autoinflammation, autoimmunity, neutrophilic dermatosis

## Abstract

Schnitzler's syndrome is a rare autoinflammatory disorder characterized by interleukin-1ß-mediated and neutrophil-dominated inflammation. Neutrophil extracellular traps (NETs) are web-like structures of decondensed chromatin, histones, and antimicrobial peptides released by neutrophils. NETs were initially described in the context of pathogen defense but are also involved in autoimmune-mediated skin diseases. Here, we assessed the role of neutrophil extracellular trap formation (NETosis) in Schnitzler's syndrome. Immunofluorescence co-staining of myeloperoxidase and subnucleosomal complex was performed on lesional skin samples from patients with Schnitzler's syndrome, other neutrophilic dermatoses (cryopyrin-associated periodic syndrome, Sweet syndrome, and pyoderma gangrenosum), urticarial vasculitis and chronic spontaneous urticaria as well as healthy control skin. Blood neutrophils from patients with Schnitzler's syndrome and controls were isolated, and NETosis was induced by phorbol 12-myristate 13-acetate (PMA). Also, NETosis of control neutrophils induced by symptomatic Schnitzler's syndrome sera, cytokines and sub-threshold PMA doses was studied. Immunofluorescence co-staining revealed widespread and substantial NET formation in lesional skin of Schnitzler's syndrome patients but absence of NETs in chronic spontaneous urticaria and control skin. Neutrophils undergoing NETosis were observed in the skin of other neutrophilic diseases too. Correspondingly, blood neutrophils from Schnitzler's syndrome patients showed significantly elevated NETosis rates compared to control neutrophils following stimulation with PMA. Increased NETosis correlated well with high levels of C-reactive protein (CRP). SchS patients with the lowest NETosis rates had persistent joint and bone pain despite IL-1 blockade. Stimulation of control neutrophils and sub-threshold PMA with sera of symptomatic Schnitzler's syndrome patients disclosed enhanced NETosis as compared to control sera. Our results suggest that the induction of NET formation by neutrophils contributes to skin and systemic inflammation and may support the resolution of local inflammation in Schnitzler's syndrome.

## Introduction

Schnitzler's syndrome (SchS) is a rare acquired autoinflammatory disease defined by recurrent urticarial rash, monoclonal gammopathy, and systemic inflammation that presents with fever episodes, muscle, bone, and joint pain (1). Until now, little is known about the exact pathophysiology of SchS. As in other autoinflammatory diseases, interleukin-1ß (IL-1β) functions as a key mediator of inflammation (2). In the skin of SchS patients, IL-1ß, and related cytokines are upregulated and produced by dermal mast cells and neutrophils (3, 4). The urticarial rash in SchS corresponds to neutrophil-rich dermal infiltrates classified as neutrophilic dermatosis. This term comprises a heterogeneous group of non-infectious inflammatory skin diseases characterized by the predominance of neutrophils in lesional skin (5). The specific role of neutrophils in SchS, however, is still ill-defined.

Neutrophils are part of the innate immune system and are responsible for the first line defense against pathogens by degranulating and secreting granule-stored antibacterial proteins as well as phagocytosis. Moreover, neutrophils are known to release neutrophil extracellular traps (NETs), which are web-like structures of decondensed chromatin, histones, and antimicrobial peptides that were initially described in the context of pathogen defense (6). NETs are also involved in various immune-mediated disorders as well as autoimmune diseases, and they contribute to the cutaneous inflammation in systemic lupus erythematosus (SLE) and psoriasis (7, 8). Recently, NETs were shown to regulate IL-1ß-mediated systemic inflammation in the autoinflammatory disease familial Mediterranean fever (FMF) (9) and were linked to arthritic inflammation in patients with gout (10).

In this study, we assessed NET formation in the lesional skin and peripheral blood of patients with SchS.

## Materials and Methods

### Patients and Patient Samples

Lesional skin biopsies (ø 3−4 mm) were obtained from symptomatic patients with SchS (based on the Straßbourg diagnostic criteria (1)) or chronic spontaneous urticaria (CSU) who were treated at the Department of Dermatology, Charité - Universitätsmedizin Berlin. At the time of the skin biopsy, patients did not receive anti-IL-1-therapy or other immunomodulatory therapies. In addition, lesional skin samples from patients with Cryopyrin-associated periodic syndrome (CAPS), urticarial vasculitis (UV), and other neutrophilic dermatoses (Sweet syndrome [SwS] and pyoderma gangrenosum [PG]) as well as skin samples from healthy control subjects who underwent bariatric, plastic or breast reduction surgery, were obtained from the Department of Surgery, Charité—Universitätsmedizin Berlin. Also, blood samples from SchS patients (canakinumab-treated within the last 8 weeks and untreated patients) and healthy donors were taken. Blood samples were derived from SchS patients who underwent skin biopsy and from additional SchS patients. The mean disease duration in SchS patients was 10.45 years when tests were performed. Skin and blood specimens from individual patients were obtained at different time points. The study was approved by the local ethics committee (EA4/005/15, EA1/007/17), and patients provided written and oral informed consent.

### Immunofluorescence Staining of Skin Samples

Paraffin sections (5 μm) of lesional skin (SchS *n* = 8, CSU *n* = 5, CAPS *n* = 3, UV *n* = 5, SwS *n* = 4, PG *n* = 4) and healthy control skin (*n* = 10) were prepared and processed for immunofluorescence staining. We stained hematoxylin eosin, myeloperoxidase (MPO) as well as subnucleosomal complex as a marker for NET formation and used DAPI (4',6-Diamidino-2-phenylindole dihydrochloride 10236276001, Roche Diagnostics Deutschland GmbH, Mannheim) for counterstaining of nuclei. MPO (1:400 392105 MAB3174 R&D Systems, Inc., Minneapolis, USA) was incubated with the secondary antibody (1:600 30 min room temperature, Alexa Fluor® 488-conjugated AffiniPure goat-anti-mouse IgG, 115-545-146; Jackson Immuno Research, West Grove, USA) and mouse normal serum 4% was added (1 h room temperature). The Alexa 488-labeled MPO antibody (overnight 4°C) was followed by incubation with Alexa 594 (1:200 30 min room temperature, Alexa Fluor® 594-conjugated Fab AffiniPure goat-anti-mouse IgG, 115-585-062; Jackson Immuno Research, West Grove, USA) labeled monoclonal mouse-anti-human-subnucleosomal complex (Histone 2A, 2B, chromatin) antibody (1:1,500 1 h room temperature). Samples of tonsil tissue served as positive controls for staining MPO and were derived from routine tissue sampling of the Department of Pathology, Charité – Universitätsmedizin Berlin. Sections omitting the primary antibody served as negative controls.

### Isolation and Stimulation of Neutrophils

Peripheral blood neutrophils from SchS patients (*n* = 12; canakinumab-treated patients *n* = 9, untreated patients *n* = 3; Table S1) and healthy controls (*n* = 12) were isolated by using a neutrophil isolation kit (MACSxpress® neutrophil isolation kit human, MACS Miltenyi Biotec GmbH, Bergisch-Gladbach, Germany, no. 130-104-434). Neutrophils were assessed for spontaneous NET formation (incubation with RPMI medium with 2% fetal calf serum for 130 min) and for NET formation after stimulation with 20 nM phorbol 12-myristate 13-acetate (PMA) for 80, 100, and 130 min as previously described (11). Neutrophils were isolated and processed in pairs of one patient and one healthy control at a time.

In addition, NET formation of healthy control neutrophils by SchS sera (10% serum concentration) from symptomatic patients (*n* = 12), combined with RPMI medium with 2% fetal calf serum as well as sub-threshold (0.05 nM) PMA doses for 130 min, was assessed. Sera from healthy individuals (*n* = 14) served as controls. Also, NET formation of control neutrophils and neutrophils from SchS patients was assessed after stimulation with IL-1β (10–50 ng/ml), IL-6 (10–100 ng/ml), IL-17 (20–100 ng/ml), or IL-8 (20–100 ng/ml) as well as with a combination of proinflammatory cytokines (IL-1β, IL-6, IL-17, IL-8) and subthreshold PMA.

### NET Detection by Immunofluorescence Staining and Quantification of NET Formation of Peripheral Blood and Skin Neutrophils

Samples were stained (immunofluorescence co-staining of DAPI and subnucleosomal complex), and microscopic images of peripheral blood neutrophils were analyzed by ImageJ as previously described (12). In brief, 5 microscopic images of each coverslip were randomly taken per experiment (100x magnification). Image files were loaded in the ImageJ software and for total cell number, the DAPI fluorescence image stack was analyzed (automatic particle analysis, 20 pixels minimum size). For neutrophils undergoing NETosis, the anti-subnucleosomal complex fluorescence image stack was counted automatically. To clearly distinguish between non-NETting and NETting cells, exposure times were calibrated to show no fluorescence signal in non-stimulated neutrophils (negative control) and full fluorescence signal of anti-subnucleosomal antibody in the PMA 130 min.-stimulated samples (positive control). We kept this threshold value of exposure time constant for each experiment. Relative NETosis rates were calculated by using the highest NETosis rate per experiment as 100%. NETosis in the skin was assessed by percentages of neutrophils undergoing NETosis per high power field. Stainings were analyzed by one blinded researcher who counted all neutrophils undergoing NETosis in five exemplified high power fields per skin sample (400x magnification). Cases of dense neutrophilic infiltration with wide-spread NETosis covering the HPF, were assessed as 100%. Exemplified stainings of NETosis were analyzed by confocal microscopy.

### Assessment of Serum Inflammation Markers and Physician Global Assessment

Sera of canakinumab-treated SchS patients (*n* = 8) were analyzed for C-reactive protein (CRP, Ref. <5.0 mg/l), serum amyloid A (SAA, Ref. <6.4 mg/l), and total blood neutrophil count (Ref. 1.5–7.7/nl) at the time of neutrophil stimulation. For analysis of S100A8/9 (Ref. <2.94 μg/ml) and interleukin-1-receptor antagonist (IL-1RA, Ref. <500 pg/ml), sera of *n* = 5 canakinumab treated patients were available. We also assessed quantitative immunoglobulins (IgM, Ref. < 2.3 g/l; IgG, Ref. 7–16 g/l). In addition, physician global assessment (PGA, categories: urticarial rash, fatigue, fever, myalgia/bone pain and arthralgia, score 0–4; maximum score: 20) was performed.

### Statistical Analysis

Statistical analysis was performed for non-parametric data by using the Mann Whitney *U*-Test. Correlation analyses for inflammation marker concentrations as well as PGA and NETosis rates were performed by Spearman's rank coefficient. For all analyses, SPSS version 22.0 and Graph Pad Prism version 6.0 were used. Statistical significance was considered by *p* ≤ 0.05.

## Results

### The Lesional Skin of Patients With SchS Is Characterized by Abundant Infiltrates of Neutrophils That Undergo NETosis

**Figure 1:**
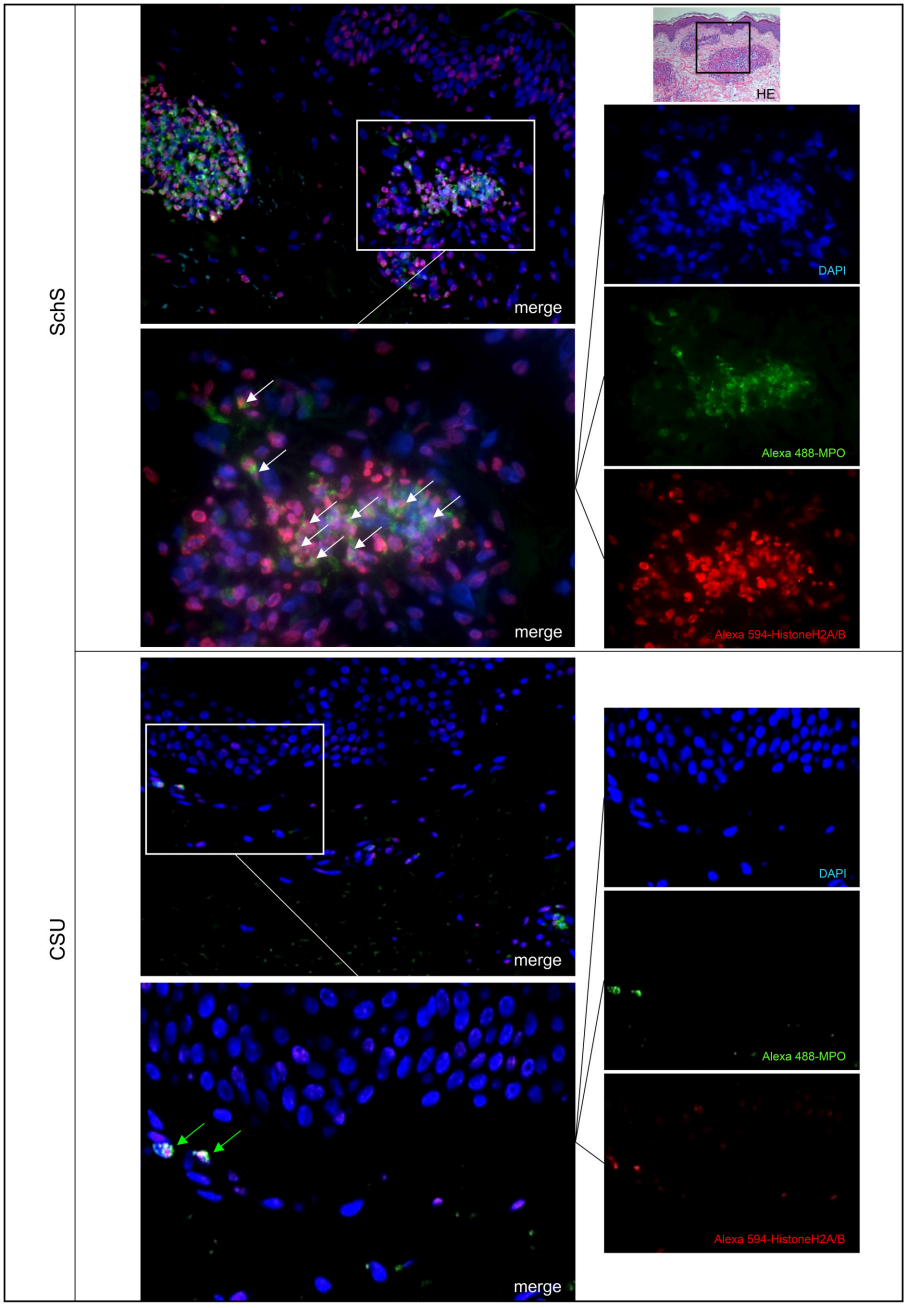
Marked NET formation in lesional skin of Schnitzler‘s syndrome (SchS) patient vs. absence of NET formation in chronic spontaneous urticaria (CSU) patient. Exemplified fluorescence images showing marked and aggregated NET formation characterized by nuclear expansion and extracellular web-like DNA-fibers (white arrows) in the lesional dermis of a SchS patient vs. absence of NETosis of neutrophils (green arrows) in lesional dermis of a CSU patient. Neutrophils (Alexa 488-MPO–green) undergoing NETosis (subnucleosomal complex Histone H2A, H2B, chromatin Alexa 594–red). Nuclei are counterstained with DAPI (blue) (original magnification 200x). Overwiew: Hematoxylin-Eosin staining of infiltrate in lesional skin of SchS patient (original magnification 200x).

**Figure 2:**
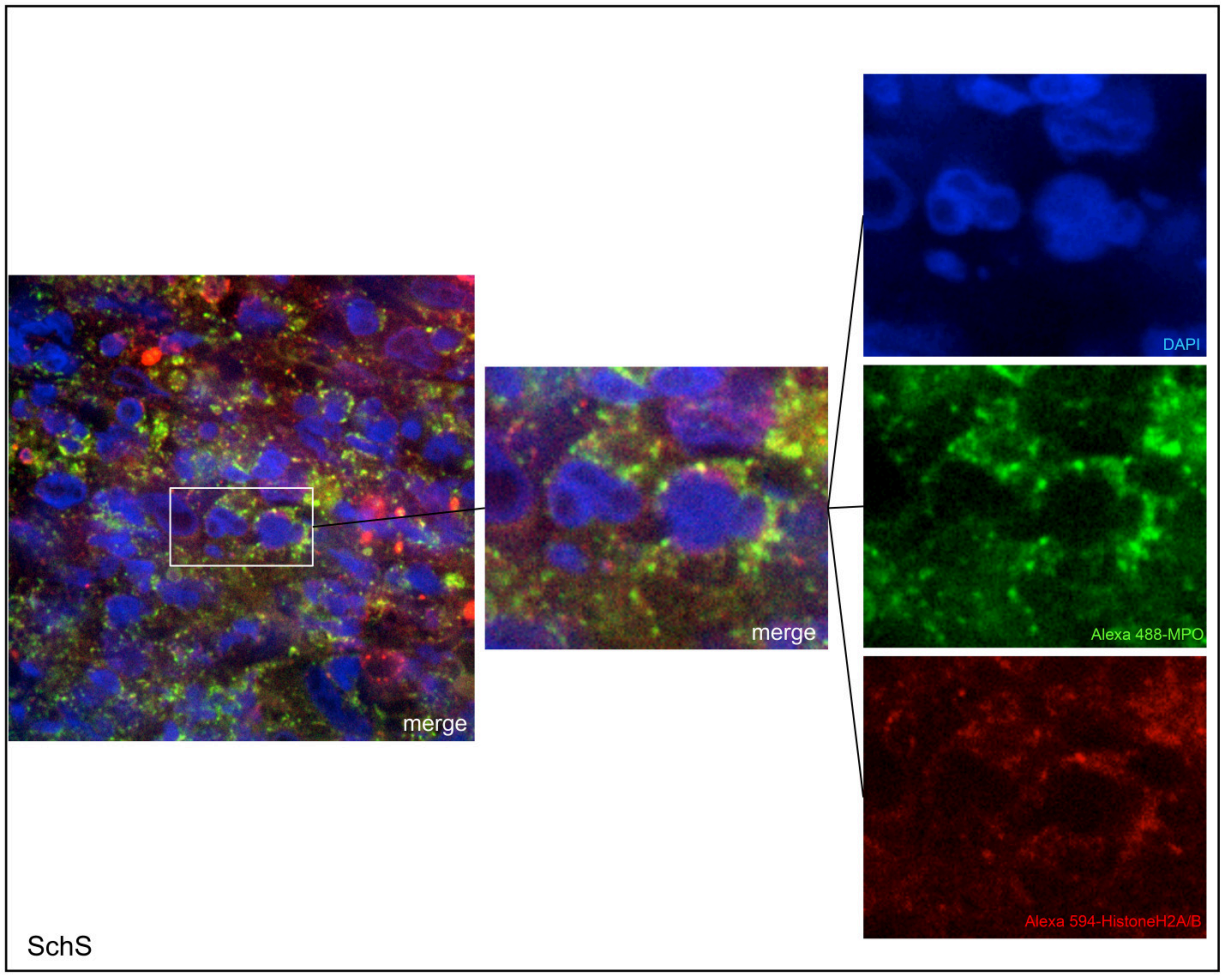
Confocal microscopic images of Schnitzler's syndrome (SchS) skin tissue. Neutrophils (Alexa 488-MPO–green) undergoing NETosis (subnucleosomal complex Histone H2A, H2B, chromatin Alexa 594–red) are demonstrated by extracelluar co-localization of subnucleosomal complex and MPO. Nuclei are counterstained with DAPI (blue) (original magnification 1,000x).

**Figure 3:**
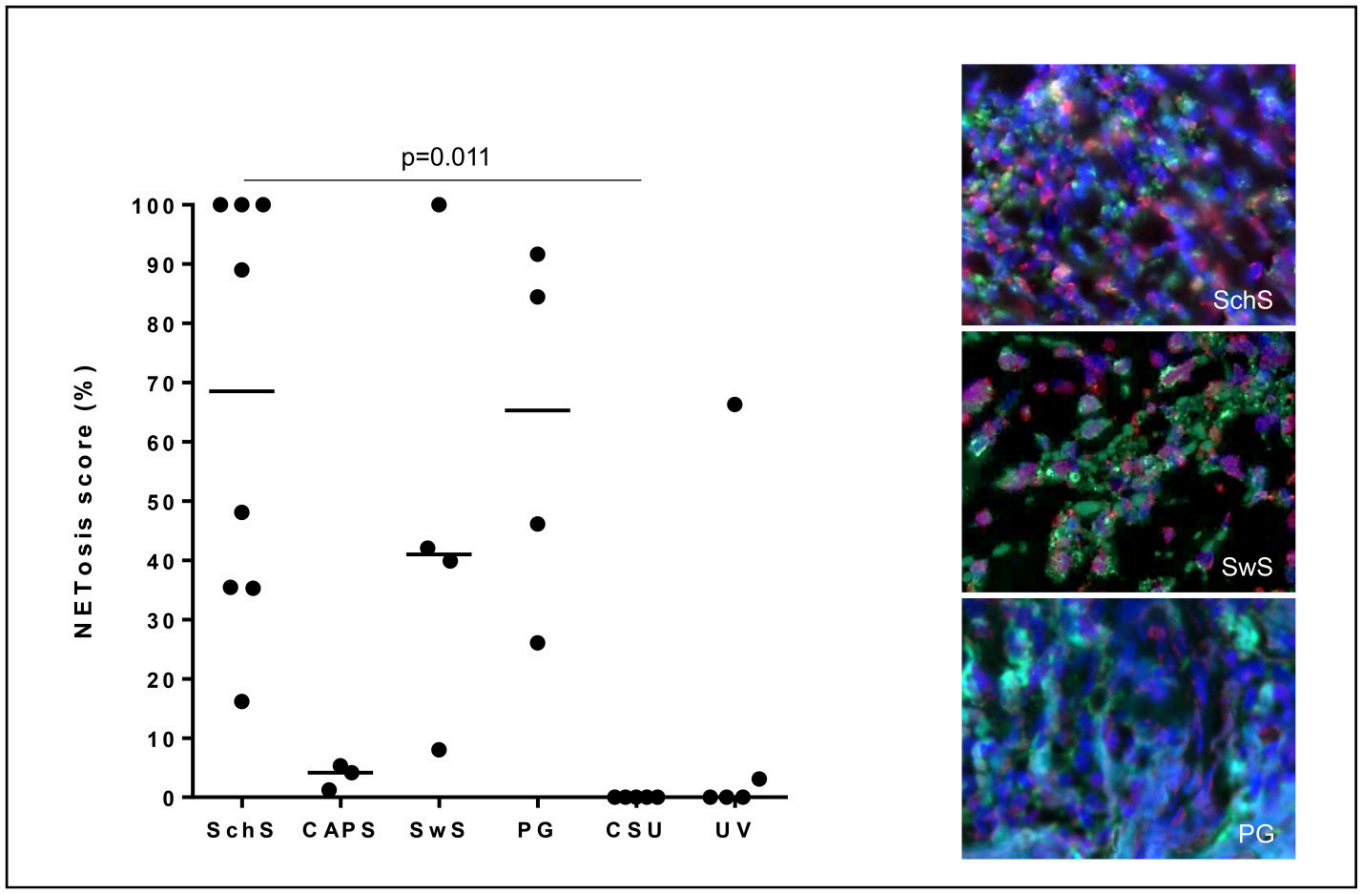
NETosis - immunoreactivity score in %: High NETosis rates in lesional dermis of Schnitzler's syndrome (SchS) patients as well as Sweet syndrome (SwS) patients and pyoderma gangrenosum (PG) patients. In lesional skin of Cryopyrin-associated periodic syndrome (CAPS) patients, only single neutrophils underwent NETosis. In *n* = 2/5 patients with urticarial vasculitis patients (UV) NETting neutrophils (single cells or aggregated NETs) were detected. Absence of NETosis rates in lesional dermis of CSU patients. Bars indicate median values. Exemplified fluorescence images showing dense neutrophilic infiltration with marked and aggregated NET formation characterized by nuclear expansion and extracellular web-like DNA-fibers in the lesional dermis of a SchS patient (top), SwS patient (middle) and PG patient (bottom). Neutrophils (Alexa 488-MPO - green) undergoing NETosis (subnucleosomal complex Histone H2A, H2B, chromatin Alexa 594 – red). Nuclei are counterstained with DAPI (blue) (original magnification 400x).

Lesional skin of SchS patients showed stronger neutrophilic cell infiltrates as compared to CSU (Figure S1). Immunofluorescence co-staining revealed marked and aggregated NET formation in lesional skin of all untreated SchS patients (*n* = 8) with highest median NETosis scores (68.54%) (Figures 1, **3**). This could be confirmed by confocal microscopy (Figure 2) which demonstrated extracellular co-localization of subnucleosomal complex and MPO in SchS lesional skin. Interestingly, marked and aggregated NET formation was also present in lesional dermal skin of patients with SwS, (median score 40.99%) and PG (median score 65.28%) (Figure 3). Only single neutrophils undergoing NETosis were observed in CAPS patients (median score 4.17%), whereas *n* = 2/5 UV patients presented with single or marked aggregated NETs (median score 0%) (Figure 3). There was an absence of NETs in the skin of CSU patients (*n* = 5) (Figures 1, 3; SchS vs. CSU p = 0.011) and healthy controls (*n* = 10) (data not shown).

### PMA-Stimulated Peripheral Blood Neutrophils of SchS Patients Show Higher NETosis Rates Than Healthy Controls

**Figure 4:**
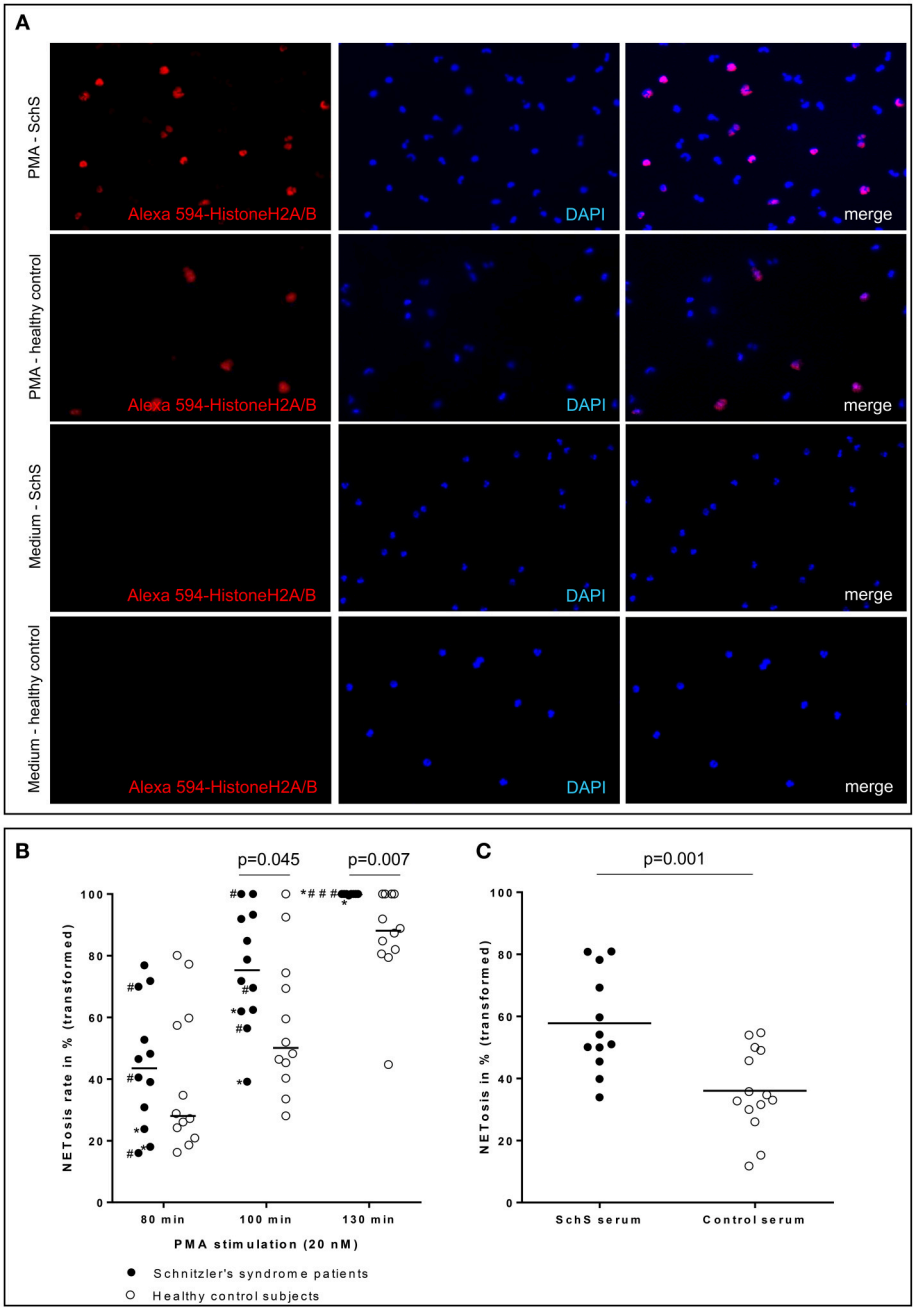
**(A)** Higher NETosis rates of blood neutrophils in Schnitzler‘s syndrome (SchS) patient vs. healthy control after 130 min of PMA stimulation: Fluorescence images of peripheral blood neutrophils of a patient with SchS compared to healthy control: NET formation of isolated neutrophils (subnucleosomal complexe Histone H2A, H2B, chromatin Alexa 594 – red). Nuclei are counterstained with DAPI (blue) (original magnification 100x) **(B)** Higher NETosis rates of neutrophils in SchS vs. healthy controls: NETosis rates in % of blood neutrophils in IL-1-inhibitor treated and untreated SchS patients (# marked untreated patients) vs. healthy controls after 80, 100, and 130 min following 20 nM PMA stimulation. ^*^patients with persistent joint/bone inflammation despite IL-1 blockade. **(C)** Higher NETosis rates after SchS serum stimulated control neutrophils compared to control serum stimulation: NETosis rates in % of control blood neutrophils after stimulation with 0.05 nM PMA and 10% serum of symptomatic SchS patients and healthy controls. Bars indicate median values.

Blood neutrophils from the majority of canakinumab-treated patients (*n* = 6/9 for 80 min and 100 min, *n* = 9/9 for 130 min) and all untreated SchS patients showed higher NETosis rates of PMA-stimulated (20 nM) blood neutrophils. NETosis rates of PMA-stimulated blood neutrophils from SchS patients increased over time compared to healthy control neutrophils with significant difference after 100 min (*p* = 0.045) and 130 min (*p* = 0.007) of PMA-stimulation (Figures 4A,B). However, considerable variability was observed in both groups and between experiments. Incubation of neutrophils with RPMI medium alone resulted in near absent NETosis rates in both SchS patients and healthy controls. Patients on IL-1 blockade (canakinumab; *n* = 6) showed similar results compared to untreated patients (*n* = 3) without significant differences between both patient groups (Figure 4B, # marked as untreated patients).

Stimulation with individual or combined proinflammatory cytokines IL-1β, IL-6, IL-8, IL-17 in different concentrations and together with PMA showed higher NETosis rates of blood neutrophils in single SchS patients, but there was no overall statistical significance between patients and healthy controls (data not shown).

### SchS Sera Induce High Rates of NETosis in Healthy Donor Neutrophils

Stimulation of healthy control neutrophils with serum of untreated symptomatic SchS patients showed near absence of NETosis rates, which is comparable to stimulation with healthy control sera as well as to stimulation with RPMI medium. Co-stimulation of healthy control neutrophils with serum of untreated symptomatic SchS patients and sub-threshold PMA (0.05 nM) disclosed significantly higher NETosis rates as compared to control sera and sub-threshold PMA (*p* = 0.001) (Figure 4C).

### High NETosis Rates Significantly Correlate With Increased Inflammation Markers

**Figure 5:**
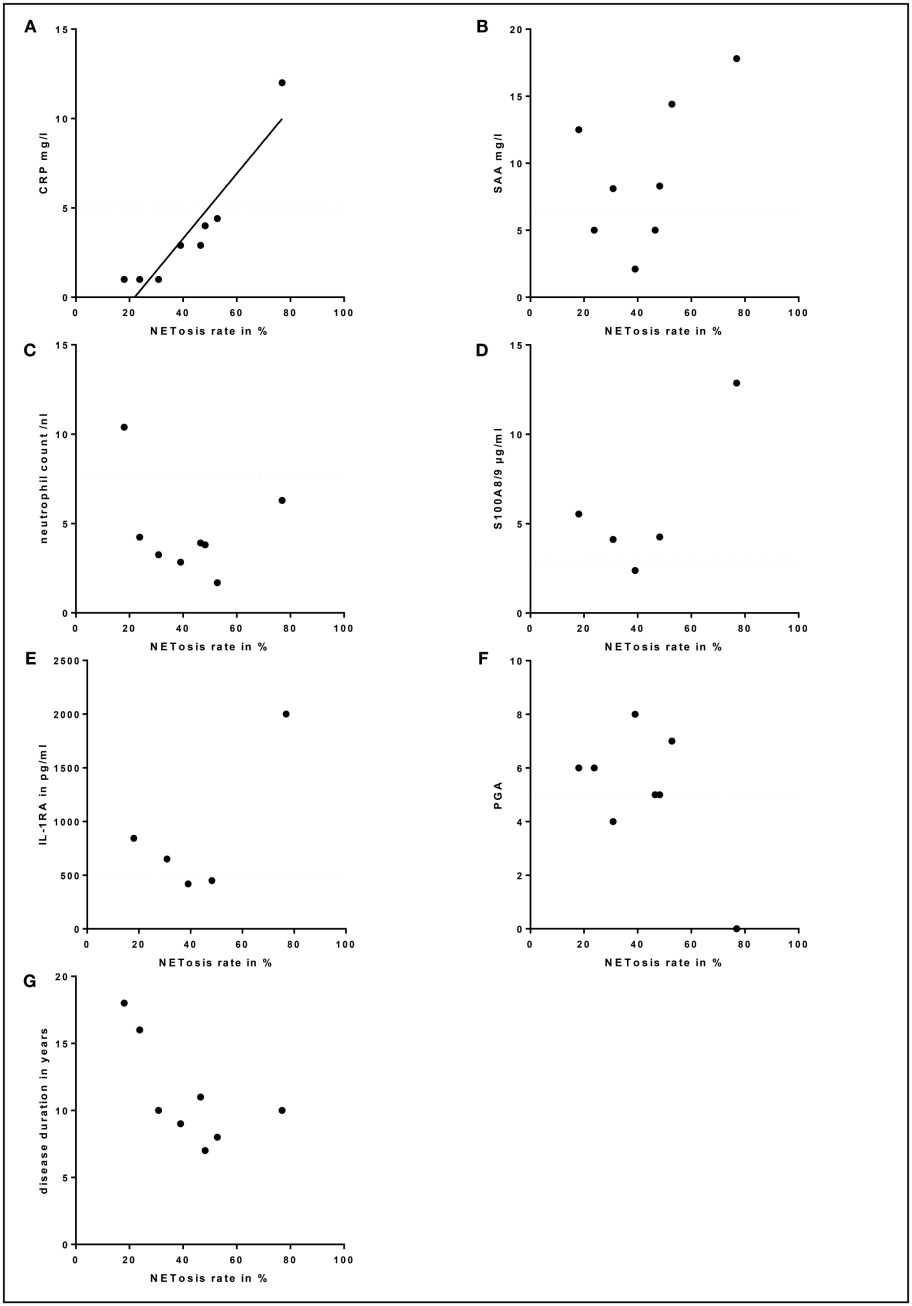
Correlation between serum markers, physician global assessment (PGA), disease duration and NETosis rates: **(A)** Correlation between high NETosis rates and high C-reactive protein (CRP) levels for 80 min PMA stimulation in Schnitzler's syndrome patients (*p* = 0.000; *r* = 0.970). No significant correlation between NETosis rates and serum levels of **(B)** serum amyloid A (SAA), **(C)** total blood neutrophil count, **(D)** S100A8/9, **(E)** interleukin-1-receptor antagonist (IL-1RA), **(F)** PGA or **(G)** disease duration was observed in Schnitzler's syndrome patients. The dotted lines indicate normal values (CRP, SAA, neutrophil count, S100A8/9, IL-1RA) and minimal disease activity (PGA).

Due to the continuous anti-IL-1 blockade in the majority of patients, inflammation markers were barely elevated. Still, a strong correlation between high NETosis rates and high CRP levels (for 80 min PMA stimulation *p* = 0.000; *r* = 0.970) was identified (Figure 5A). In contrast, no significant correlation between NETosis rates and markers of subclinical inflammation (SAA, S100A8/9, IL-1RA), neutrophil count, disease duration or clinical disease activity assessed by PGA was observed (Figure 5). Also, we could not detect a significant correlation between NETosis rates of symptomatic SchS patients and immunoglobulin levels. The two SchS patients with the lowest NETosis rates had persistent joint and bone pain despite IL-1 blockade (Figure 4B, ^*^ marked patients with persistent joint and bone pain). Vice versa, 2 out of 3 patients with high NETosis rates had only mild bone pain/arthralgia.

## Discussion

Our results demonstrate increased amounts of neutrophils undergoing NETosis in lesional skin and peripheral blood of SchS patients as compared to CSU and/or healthy controls. To the best of our knowledge, this is the first study which reports NETosis in SchS. Furthermore, it supports the significance of NET formation in autoinflammatory diseases as previously described for FMF and gout (9, 14).

Neutrophils undergoing NETosis in lesional skin of SchS patients and their absence in CSU patients may explain the clinical observation of more solid and stable wheals with longer duration in autoinflammatory patients compared to urticarial lesions in CSU patients (15). In contrast to SchS, lesional skin of CSU patients usually shows rather sparse lymphocytic infiltrates and fewer neutrophils (4). The spectrum of UV varies from only mild urticarial lesions to severe disease with systemic manifestations which could explain the heterogeneity of UV patients regarding the occurrence of NETs in lesional skin. Clinical phenotypes of neutrophilic dermatoses include pustular, urticarial, plaque-like, and ulcerous lesions. All of them are commonly associated with systemic diseases such as hematologic, autoimmune and autoinflammatory entities (5). The presence of NETs in SwS and PG suggests that NETosis contributes to the pathogenesis of other neutrophilic dermatoses as well. This is underscored by a very recent publication showing that enhanced NET formation and decreased NET degradation contribute to the inflammation in the hereditary autoinflammatory disease Pyogenic arthritis, pyoderma gangrenosum and acne (PAPA) syndrome (16). Although neutrophilic dermatoses are clinically heterogenous disorders linked to either autoimmune or autoinflammatory pathways, they share the activation of neutrophils that could be elicited via alterations in homeostasis or chemotaxis of neutrophils (17).

NETosis was previously associated with skin inflammation in psoriasis and SLE (7, 8). Psoriasis is characterized by epidermal hyperproliferation and neutrophil infiltrates invading the epidermis. As expected, NETosis was primarily observed in the epidermis next to keratinocytes (7), whereas in SchS epidermal involvement is missing, and neutrophil infiltrates undergoing NETosis are dermally located as can also been seen in SLE (8).

A dual role for NETs was implicated in the pathophysiology of inflammatory diseases. In prototype autoimmune disease SLE, NETs induce the expression of double stranded DNA (dsDNA) and LL-37, resulting in externalization of autoantigens and immunostimulatory proteins (8). These may contribute to an antigenic source for autoantibodies that promote the inflammation and damage of endothelial cells by synthesis of proinflammatory cytokines or type I interferons in SLE (8). On the other hand, NETs may limit the inflammation as suggested for autoinflammatory disorders gout and FMF (9, 14). Aggregated NETs in gout were shown to contribute to the resolution of inflammation by degrading cytokines and chemokines via serine proteases (14). In FMF, neutrophils were demonstrated to release IL-1ß through NETs, but they also downregulated further NETosis and thereby resolved FMF attacks via a negative feedback mechanism (9).

**Figure 6:**
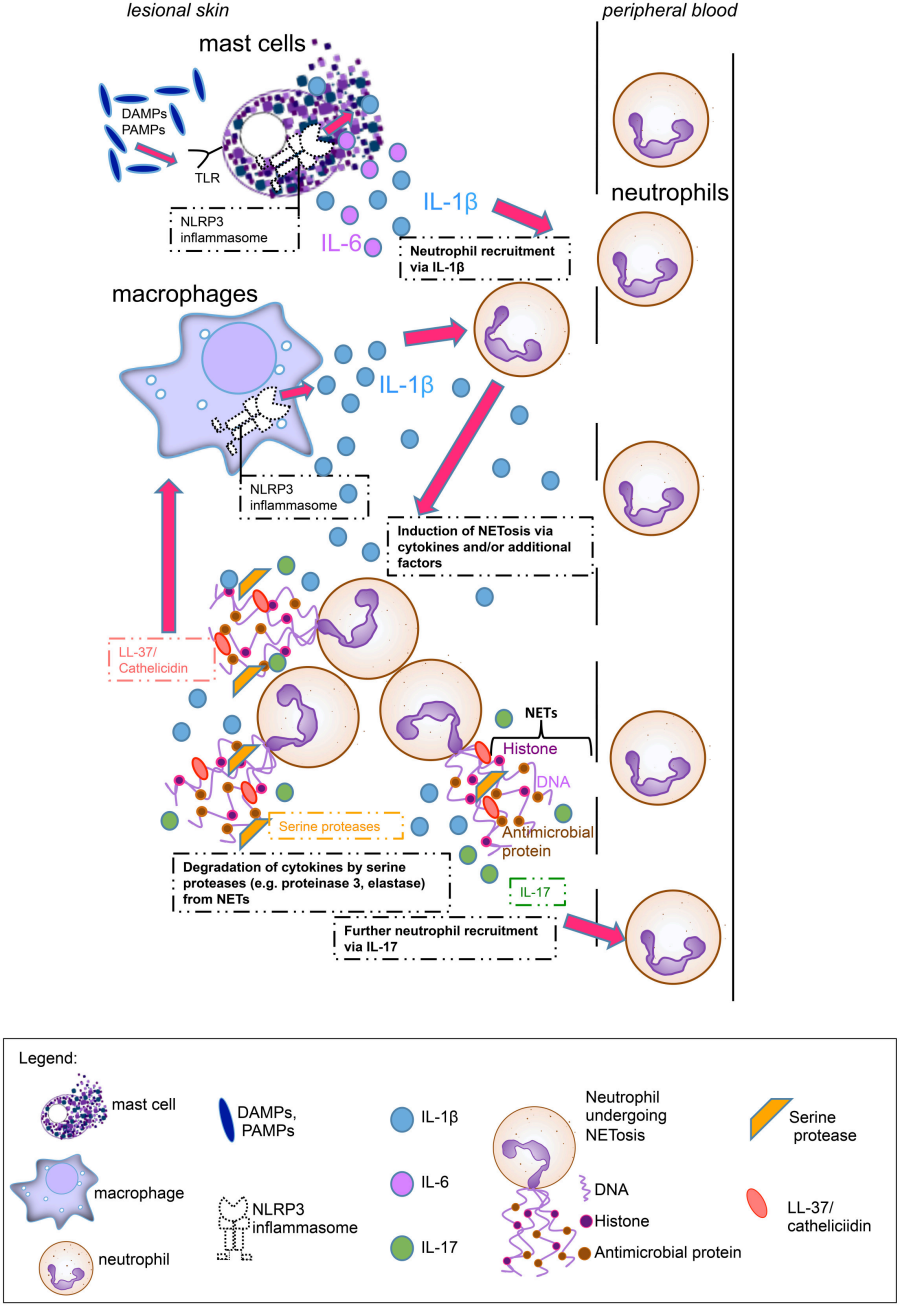
Main hypothesis on the pathophysiological role of neutrophil extracellular traps (NETs) in Schnitzler's syndrome: Mast cells hypersensitive to damage-associated molecular patterns (DAMPs) or pathogen-associated molecular patterns (PAMPs), are activated via Toll-like receptors (TLRs) and produce Interleukin (IL)-1ß and IL-6. Cytokine production by mast cells is presumably mediated via the NLRP3 inflammasome. These proinflammatory cytokines lead to neutrophil recruitment from peripheral blood. Cytokines and other serum factors induce initial NET formation in the skin. During NETosis, IL-17 and LL-37 (cathelicidin) are secreted. IL-17 induces further neutrophil recruitment, while LL-37 activates the NLRP3 inflammasome in macrophages to produce further IL-1ß, which again recruits neutrophils. Additional NETting neutrophils aggregate and secrete serine proteases such as proteinase 3 and elastase which degrade cytokines (e.g., IL-1ß) and limit inflammation.

NETosis rates in psoriasis patients correlated well with clinical disease activity (7). Although this was not the case in our study, we observed a positive correlation between high NETosis rates and increased inflammatory marker CRP in SchS. We hypothesize that the systemic inflammation drives NET formation in SchS. These NETs may exert both pro- and anti-inflammatory functions. NETosis could stimulate aberrant NLRP3 inflammasome activation in SchS monocytes and macrophages via cathelicidins and thereby enhance the release of inflammatory cytokines followed by further NETosis as shown for SLE (18). Also, NETs may support the resolution of inflammation by degrading cytokines locally in SchS (Figure 6). As in gout inflammation, high amounts of aggregated neutrophils were found in lesional tissue of SchS patients. The inflammation-limiting ability of NETosis via cytokine degradation could explain the observation that SchS patients with low NETosis rates suffer from persistent chronic joint and bone inflammation as degradation of cytokines may not be sufficient in these patients.

In lesional skin of patients with prototype autoinflammatory urticarial syndrome CAPS, we observed only single neutrophils undergoing NET formation. This finding may indicate a pathophysiological link of NET formation and SchS-specific paraproteinemia. Nevertheless, we could not show a significant correlation between NETosis rates and immunoglobulin levels, and there is no evidence for a link between paraproteinemia and NET formation in other diseases associated with paraproteinemia. However, aberrant inflammasome component expression is described in both SchS and multiple myeloma (19, 20).

The pathophysiologic mechanisms of how neutrophils are activated to undergo NETosis locally in the lesional tissue as well as systemically in the peripheral blood of SchS patients, are unclear. Our results of higher NETosis of donor neutrophils induced by SchS serum compared to healthy serum are in line with the findings in psoriasis and support the existence of a potential serum factor inducing NETosis (7). Several proinflammatory cytokines, including IL-1ß, IL-6, and IL-17 were shown to be elevated in the serum of different inflammatory conditions such as sepsis, diabetes and rheumatoid arthritis and to induce enhanced NETosis of peripheral neutrophils in these disorders (12, 21, 22). These cytokines are known to be upregulated in SchS as well (3, 4). Interestingly, mast cells induce neutrophil recruitment and were shown to be a source of IL-1β and IL-6 in lesional skin of SchS patients (3, 4). Whether these proinflammatory cytokines e.g., produced by mast cells or even neutrophils themselves contribute to NET formation locally in lesional skin of SchS patients remains to be elucidated. However, none of these cytokines, whether alone or in combination, could induce NETosis of peripheral neutrophils in our study, and we are assuming that additional factors are needed to stimulate neutrophils to form NETs in SchS.

Limitations of our study comprise the relatively small patient number, which is explained by the rarity of SchS and the heterogeneity of individual experiments showing considerable variability.

In conclusion, our results suggest that skin and systemic inflammation in SchS are associated with neutrophils undergoing NETosis. These may exert pro-inflammatory or in the case of aggregated NETs anti-inflammatory properties. For the future, evaluation of drivers of NET formation in autoinflammatory diseases are essential in order to better understand disease mechanisms. Also, these studies may help to identify therapeutic targets that promote cytokine degradation and resolution of inflammation via enhancing aggregation of NETs.

## Data Availability

All datasets generated for this study are available from the corresponding author upon reasonable request.

## Author Contributions

HB designed and performed the experiments, analyzed the data, and prepared the manuscript. JS designed the experiments and provided intellectual assistance. JW performed the experiments. SH performed confocal microscopy. MM gave critical input to the manuscript. KK took care of the patients, designed the experiments and prepared, and revised the manuscript. All authors read, reviewed, and approved the final manuscript.

### Conflict of Interest Statement

MM received fees from Novartis for consultancy on topics outside of those of this publication. KK received honoraria and/or consultancy fees from Novartis, Roche and SOBI on topics outside of those of this publication. The remaining authors declare that the research was conducted in the absence of any commercial or financial relationships that could be construed as a potential conflict of interest.

## Supplementary Material

Table S1 Main clinical, biological, and therapeutic features of Schnitzler's syndrome patients included in this study. ^*^Patients were untreated when NETosis rates were assessed. #NETosis rates were assessed in untreated and treated patients.

Figure S1 Hematoxylin-Eosin staining of infiltrates in lesional skin of patients with Cryopyrin-associated periodic syndrome (CAPS), Schnitzler's syndrome (SchS), Sweet-Syndrome (SwS), Pyoderma gangrenosum (PG), chronic spontaneous urticaria (CSU), urticarial vasculitis (UV), and healthy controls (original magnification 50x, 200x). Images are published in part in Bonnekoh (13).

## Abbreviations

CAPS: Cryopyrin-associated periodic syndrome
CRP: C-reactive protein
CSU: Chronic spontaneous urticaria
DAPI: 4′,6-Diamidino-2-phenylindole dihydrochloride
FMF: Familial Mediterranean fever
IL: Interleukin
IL-1RA: Interleukin-1-receptor antagonist
MPO: Myeloperoxidase
NETs: Neutrophil extracellular traps
NLRP: Nucleotide binding domain like receptor protein
PG: Pyoderma gangrenosum
PGA: Physician global assessment
PMA: Phorbol 12-myristate 13-acetate
SAA: Serum amyloid A
SchS: Schnitzler's syndrome
SLE: Systemic lupus erythematosus
SwS: Sweet syndrome
UV: Urticarial vasculitis.
