# Supplementary material for: Skin and Systemic Inflammation in Schnitzler's Syndrome Are Associated With Neutrophil Extracellular Trap Formation
Source: Front Immunol. 2019 Mar 22;10:546. doi: 10.3389/fimmu.2019.00546 (PMC6438918; doi:10.3389/fimmu.2019.00546)
Supplement: Table S1 — Main clinical, biological, and therapeutic features of Schnitzler's syndrome patients included in this study. *Patients were untreated when NETosis rates were assessed. #NETosis rates were assessed in untreated and treated patients. [file Table_1.pdf]

**Patients**	**Age in years**	**Gender**	**Disease duration in years**	**overall disease severity**	**Paraprotein IgM Ref. <2.3 g/l**<br><br>**IgG Ref. 7-16 g/l**	**Treatment responses**
**1**	59	female	10	moderate	IgM kappa<br>5.53	good response to canakinumab
**2**	75	female	9	severe	IgM kappa<br>10.39	good response to canakinumab
**3**	56	male	7	moderate	IgM kappa<br>7.88	complete response to canakinumab
**4**	55	female	18	severe	IgG kappa<br>3.52	moderate response to canakinumab and anakinra
**5**	75	male	10	moderate	IgM kappa<br>15.24	complete response to canakinumab
**6**[#]	51	female	16	moderate	Not available	complete response to canakinumab, and anakinra
**7***	65	female	1	mild	IgG kappa<br>7.61	lost to follow-up
**8***	69	male	9	mild	IgM kappa<br>5.2	complete response to canakinumab
**9**	71	male	16	severe	IgM kappa<br>6.6	moderate response to canakinumab
**10**	61	female	8	severe	IgG kappa<br>16.07	moderate response to canakinumab
**11**	67	male	11	moderate	IgM kappa<br>9.43	complete response to canakinumab

Table S1
