# Supplementary figures and images for: Skin and Systemic Inflammation in Schnitzler's Syndrome Are Associated With Neutrophil Extracellular Trap Formation
Source: Front Immunol. 2019 Mar 22;10:546. doi: 10.3389/fimmu.2019.00546 (PMC6438918; doi:10.3389/fimmu.2019.00546)

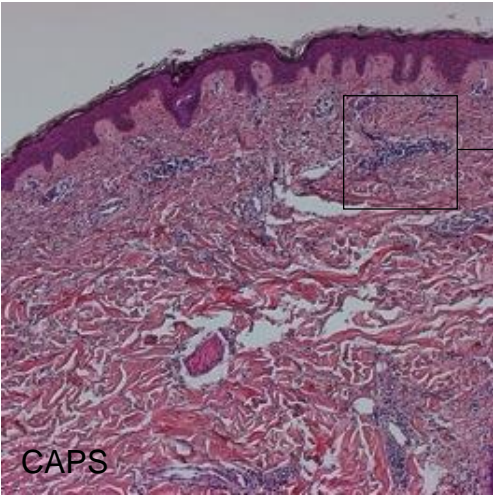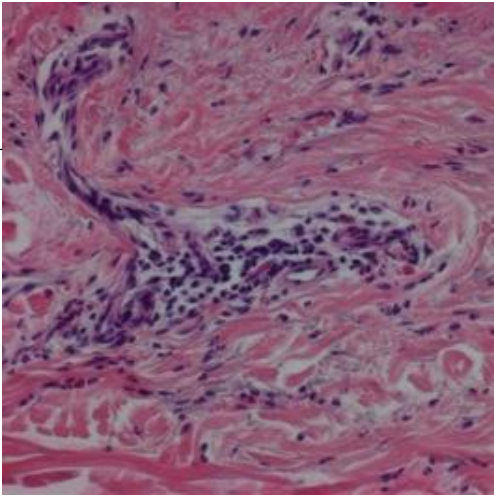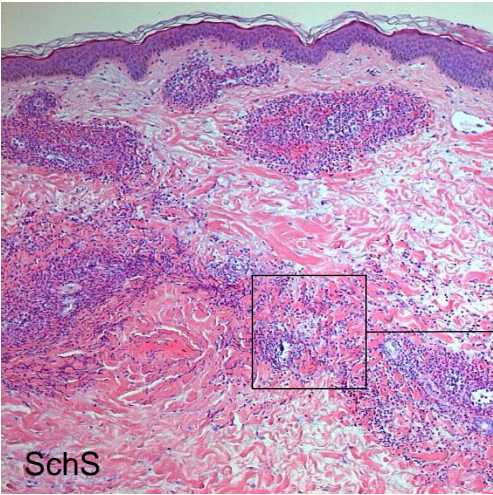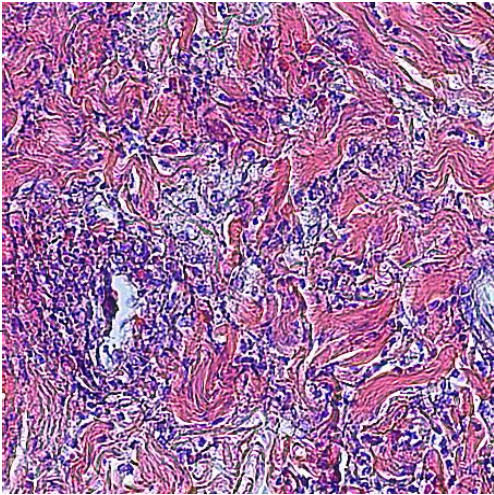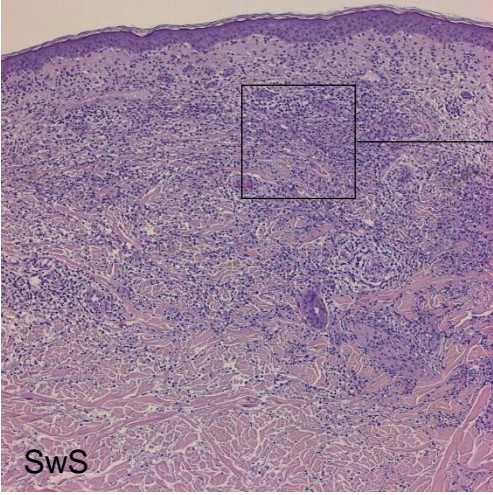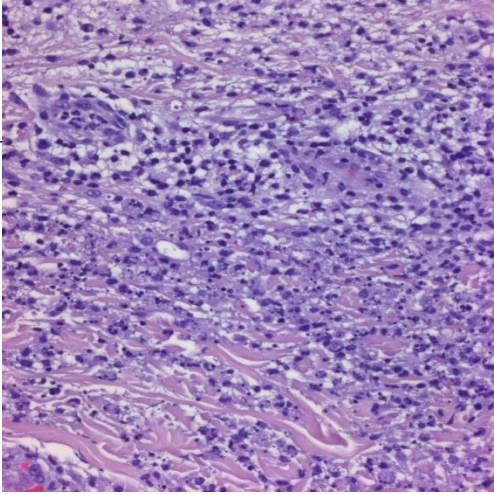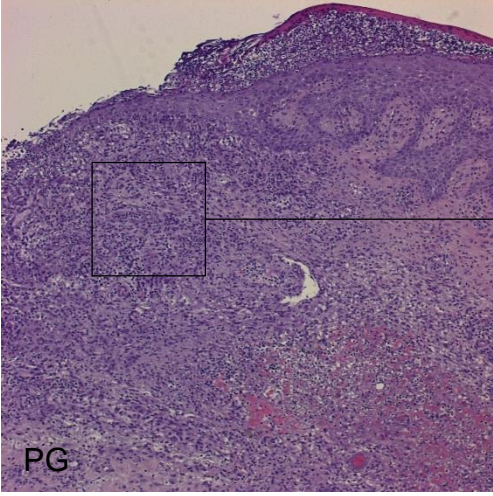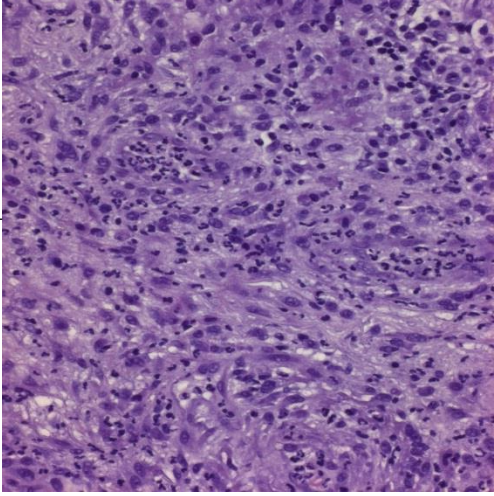

Figure S1

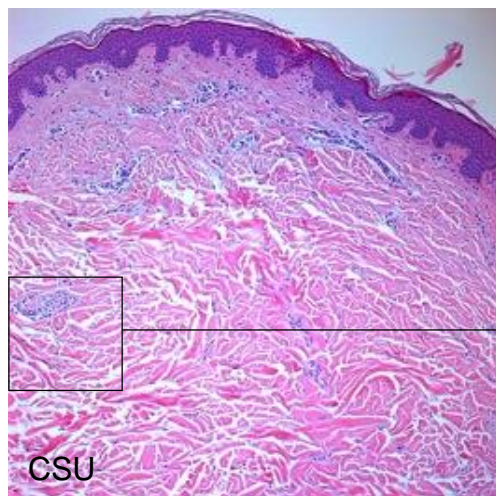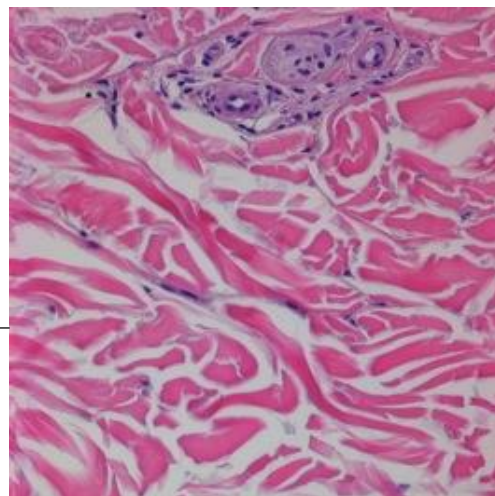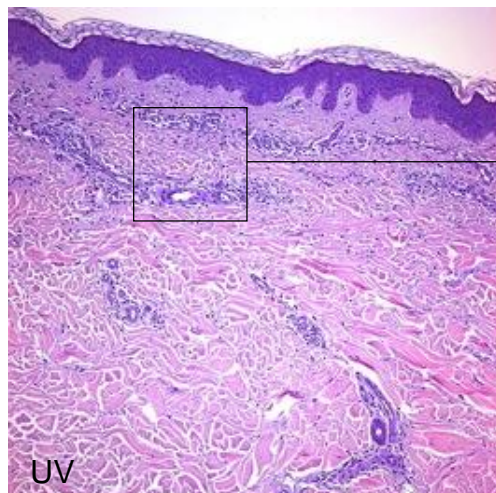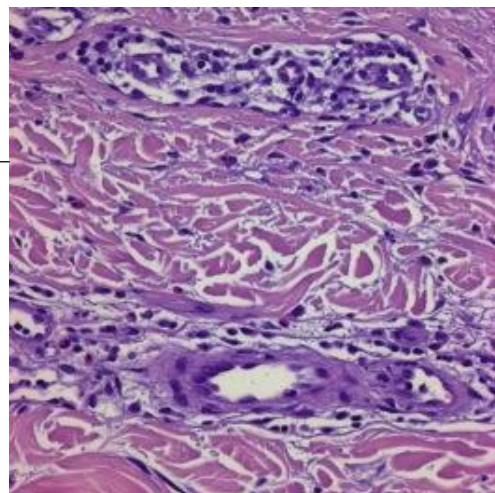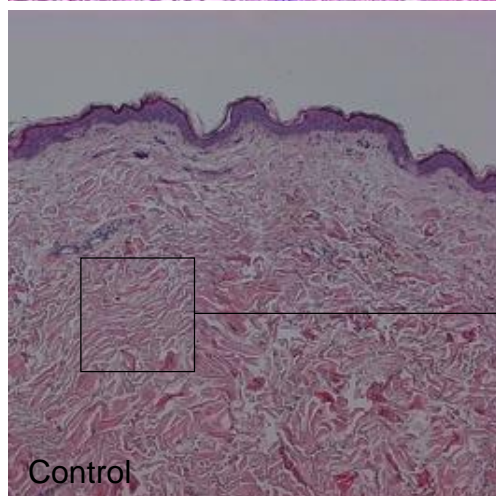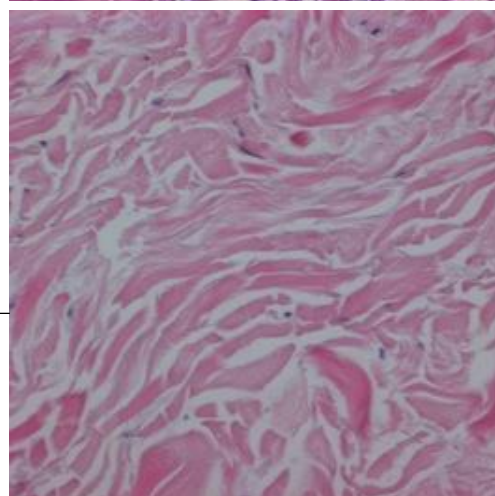

Figure S1

Supplement: Figure S1 — Hematoxylin-Eosin staining of infiltrates in lesional skin of patients with Cryopyrin-associated periodic syndrome (CAPS), Schnitzler's syndrome (SchS), Sweet-Syndrome (SwS), Pyoderma gangrenosum (PG), chronic spontaneous urticaria (CSU), urticarial vasculitis (UV), and healthy controls (original magnification 50x, 200x). Images are published in part in Bonnekoh (13). [file Image_1.pdf]
